# Posture, proximity, and positionality: the power of community engaged service-learning in public health leadership education

**DOI:** 10.3389/fpubh.2025.1605757

**Published:** 2025-07-02

**Authors:** Jocelyn C. Chu, Abrania Marrero

**Affiliations:** ^1^Office of Educational Programs, Harvard T.H. Chan School of Public Health, Boston, MA, United States; ^2^Department of Nutrition, Harvard T.H. Chan School of Public Health, Boston, MA, United States

**Keywords:** community engaged learning, service-learning, public health education, health equity, humility, critical reflection, public health leadership

## Abstract

**Introduction:**

Public health leadership is a call to action, drawing us nearer to the individuals and communities burdened by health disparities and social injustice. Reimagining public health leadership to center health equity entails collective and community engaged applied practice, premised on humility, shared power, and life-long learning. Public health education has a unique imperative to offer experiential, transformative opportunities for students to learn and practice more adaptive approaches to public health action.

**Methods:**

The Community Engaged Learning Fellowships at Harvard T. H. Chan School of Public Health support students and postdoctoral trainees to partner with organizations and implement co-designed community engaged projects. Through a cohort learning model, fellows engage in field-based projects with learning objectives centered on assuming the posture of a learner, proximity to community partners, and critical reflection around positionality and broader structural determinants of health. Qualitative data evaluating fellows’ learnings were collected. Responses from nine cohorts of fellows over 6 years (2018–2024) were analyzed thematically to reveal key insights into fellows’ overall learning experiences.

**Results:**

Fellows expressed the need to enact humility, relationality, and the centering of community expertise as values that could shift power away from themselves and toward community-identified priorities and decision-making. Embodying attitudes of authenticity and flexibility was central to fellows’ perceptions of this more equitable community engaged practice, ensuring that project goals, timelines, and unanticipated challenges, for example, reflected their partners’ agendas and lived experiences. Importantly, these deferential forms of service still contributed meaningfully to fellows’ learnings. Often, instead of holding on to preconceived project expectations, fellows practiced the skill of listening to learn and trust building to identify co-creative forms of problem-solving.

**Discussion and implications:**

Our findings reinforce the importance of community engaged service-learning as a pedagogical strategy in public health leadership development, one that instills values, attitudes, and skills that are premised on leaders becoming “learners.” Community engaged service-learning cultivates a practice that decenters self-interests, uplifts community expertise, values authentic relationships, and promotes more collective forms of decision-making. Ensuring these opportunities are available in graduate education can foster commitments to community partnerships premised on equity and service in future leaders.

## Introduction

Global public health challenges in the 21st century are complex and deeply interconnected, including the rise of non-communicable diseases (e.g., heart disease, cancer), climate change impacts, substance misuse, poor health systems infrastructure, antimicrobial resistance, infectious diseases, global pandemics, and unaddressed mental and maternal and child health concerns (1–3). Social determinants of health (i.e., the conditions and systems in which people are born, grow, work, live, and age) are well-documented drivers of the unequal burdens placed on certain populations and speak to the importance of considering the context and systems affecting such health outcomes within specific communities (4, 5). Aware of people and place, widening health inequities require multifaceted solutions and point to the need for real-world community engagement in public health leadership and practice.

Current pedagogical models of public health leadership development focus on the adaptive nature of leadership and its orientation toward action. Heifetz’s theoretical framework, for example, views leadership not as a trait or position, but as a process of mobilizing people to address complex challenges (6). Similarly, Marshall Ganz defines leadership as the “responsibility for enabling others to achieve purpose under conditions of uncertainty” (7). Learning to accept chaos -the unfamiliar, the ambiguous, and the paradoxical event- is essential for leadership in public health (7). Writing in one of the first commentaries on public health leadership in the 21st century, former Assistant Secretary for Health and Human Services Howard Koh notes the nuanced capacity leaders must have to make multidimensional decisions in the face of complexity and uncertainty (8). Rather than remaining inert under the auspices of a title or position, contemporary public health leaders are “collaborative servant leader[s] who knit and align disparate voices together behind a common mission” (8).

Public health education has a unique imperative to offer graduate students and postdoctoral trainees training opportunities to sharpen skills in problem-solving and more collective, creative, and agentic approaches to public health action (9, 10). Experiential learning through community engagement that extends beyond the classroom and into field-based work can equip students and, ultimately, public health leaders and practitioners with the practical skills and deepened learnings needed to contribute effectively to the priorities of communities most impacted by health inequities (11).

Service-learning is a form of transformative experiential education. Learning in community engaged service-learning occurs through iterative cycles of action and reflection (12), encouraging students to work directly with community members, translate theoretical frameworks into applied practice, and critically reflect on personal experiences to deepen understandings of themselves and their roles in achieving community-identified objectives (13). Service-learning embodies democratic ideals of civic engagement and community involvement, aiming to benefit both learners and communities, and offers a framework by which students engage in field work not only focused on project tasks but through a broader process of listening to learn, critical reflection, and attention to contexts and social systems (14). Without community engaged service-learning opportunities, public health leaders at all levels (e.g., policymakers, researchers, civil society actors) may fail to understand, commit to, and carry out a more proximal, deferential sense of public health practice.

Based on a critical service-learning framework (15), Community Engaged Learning (CEL) at the Harvard T. H. Chan School of Public Health combines the practice of community engagement with a service-oriented intention for students take on the role of a “learner” in the field. Distinct from internships, community service, or volunteerism, CEL moves toward a more “critical approach,” one that emphasizes the redistribution of power among all members of the CEL partnership; development of authentic relationships through reflexive, collective, and relational approaches; and work from a systems and social change perspective (16, 17). By combining critical pedagogical frameworks with service-learning (which is often implemented only at the undergraduate level), the Harvard Chan CEL fellowships offer a distinct approach to community engagement for post-graduate students and trainees at the cusp of careers in public health leadership. In this article, we describe the pedagogical framework of CEL programming at Harvard Chan and share our learnings from 6 years of program development and its implications for leadership development within public health training and education.

## Methods

### Program description

CEL fellowships at Harvard Chan support graduate students and postdoctoral fellows across the school and in all degree programs to implement domestic and international field-based projects through community-based, collaborative co-design and development (Table 1). The program aims to have students: (1) address community-identified needs and priorities, working in partnership with community members and partner organizations, (2) cultivate a community-centered approach to public health research and practice, including a deeper commitment to addressing structural social determinants of health, and (3) develop and strengthen reciprocal institutional relationships between Harvard Chan and community organizations in which the engaged learning projects take place. The fellowship is extracurricular (i.e., non-credit bearing) but can support practicum experiences (e.g., Applied Practice Experiences), dissertation projects, and/or other academic requirements.

**Table 1:** Distribution of community engaged learning fellows at Harvard T.H. Chan School of Public Health by degree program or position and project location.

Degree program or position	Count of fellows (%) (*n* = 200)^1^	Project location	Count of fellows (%) (*n* = 200)^1^
MPH-45^2^	55 (28%)	Greater Boston area	62 (31%)
MPH-65^2^	68 (34%)	United States (Other)	34 (17%)
SM^3^	25 (13%)	International	104 (52%)
PhD^4^	26 (13%)		
DrPH^4^	19 (9.5%)		
Postdoctoral fellow^5^	7 (3.5%)		

1 Data representative of fellows from all cohorts, one per semester, from Spring 2018 to Spring 2024.

2 Both Master of Public Health (MPH) degree programs at Harvard Chan—a 45-credit (for individuals with ≥ 5 years of public health experience) and a 65-credit (for those with ≥ 2 years of full-time public health experience) program—have a required applied practice experience.

3 The Master of Science (SM) degree primarily prepares students for careers in public health research.

4 The PhD doctoral program primarily prepares students with research and analytic skills needed to address complex, large-scale public health issues. The DrPH doctoral program primarily prepares students for leadership roles in public health policy, community-based work, and health care.

5 Postdoctoral fellows are research-oriented trainees with appointments throughout Harvard Chan.

Through a cohort-based model of learning, CEL fellows are part of an interdisciplinary learning community where they can share observations, questions, and challenges as well as reflect collectively on their approaches to community engaged research and practice with peers. Each semester, the program admits approximately 13–18 fellows through a competitive application process, a cohort size that has remained relatively stable over time. As a comprehensive program, CEL fellows commit to participating actively in preparing for and reflecting on community engagement, both with fellow cohort members and communities of practice in the field. Learning begins at orientation, iterates in the field, and is reflected on during debrief as fellows return to campus (Table 2). The cohort meets on campus for two mandatory, 1.5-h sessions for orientation and debrief. Fellows are also invited to participate in smaller peer groups virtually (organized by project topic, design, and/or location) as well as an informal, in-person dinner after returning from the field. All meetings are facilitated by program staff from the Office of Educational Programs. While the fellowship typically supports the implementation of specific, time-bound project tasks over winter and summer terms (for Fall and Spring cohorts, respectively), students are also encouraged by program staff to identify partners in advance, prepare for their projects during the semester, and plan for project transition strategies once field-based work ends. Importantly, a map-based database of previous partner organizations is provided to prospective fellows to foster continuity in the support received by communities involved in the fellowship over the years.

**Table 2:** Community engaged learning fellowship: framework and learning objectives.

Orientation (learning objectives)	Take on the posture of a learner, including integrating diverse ways of knowing (e.g., storytelling) beyond academic knowledge generation.
	Seek proximity to people and place, to build relationships and situate the context (e.g., systems, structures, root causes) of the public health issue of interest.
	Practice critical reflection, unearthing personal assumptions and positionality, making meaning out of experiences, and transforming learning into practice.
In the field	Learn as an iterative practice of reflection and action, including sense-making through personal writings in journals received at orientation.
	Take a “Pause in the Field,” submitting an online progress report with open-ended prompts to critically reflect on field experiences as they relate to the “Three P’s.”
	Share knowledge and experiences gained with other fellows to foment community of learners in the cohort via group text-based messaging.
Debrief	Share stories of community engaged learning experiences with other current fellows and consider the “retelling” of these stories within broader contexts of community and collective power for transformative learning.
	Share lessons learned in the field with future fellows through two open-ended, short answer written prompts (e.g., “What did you wish you knew before your community engaged learning experience?”)

At Harvard Chan, CEL invites fellows to take on the *posture* of a learner, build relationships and gain a deeper understanding of systems and contexts through *proximity* to community partners in the field, and interrogate their *positionality* so as to understand how their intersecting identities might shape their understanding and engagement with their projects. Practicing critical reflection is emphasized to help fellows bring awareness to and challenge their assumptions, engage more equitably with community members, and make personal meaning of their experiences to facilitate transformative learning.

Fellows are introduced to the main learning objectives of the program which have, at their core, these “Three P’s” (i.e., posture, proximity, and positionality) during orientation and are prompted to reflect on their experiences through these community engaged concepts in the field and at debrief (Table 2). They are also prompted to consider tenets of equitable, community-engaged co-design from project conceptualization to dissemination. This includes extensive, iterative, and flexible communication with partners so that projects address community-identified priorities (including those which the organization may have had limited time or resources to work on), are embedded within organizational processes, explicitly address potential power imbalances through transparency and accountability (e.g., data-sharing agreements), and dedicate funding toward elements that directly recognize community expertise (e.g., honoraria). This framework was developed through an engaged pedagogical practice and is now central to assessing learning outcomes in the fellowship program (18). The program’s framework was formalized and produced in the form of a handbook in Spring 2024 (Supplementary Figure 1).

### Qualitative evaluation

Qualitative endpoint data evaluating students’ overall learnings within the CEL framework as a synthesis of community engagement concepts introduced during orientation, their practical implementation in the field, and iterative reflections and reframing during the debrief and throughout the fellowship was collected at the end of the debrief through writing exercises asking fellows two open-ended questions: (1) “What did you wish you knew before your CEL experience?” and (2) “What advice would you give to the next cohort of fellows?” This exercise aimed to capture, in short form, the narrative by which fellows made sense of the knowledge and skills they gained as a result of their field experience (experiential learning) and critical reflection (transformative learning) (19, 20). The two prompts were unstructured, so as to capture information inductively, and framed to elicit peer-to-peer knowledge sharing, a reflective process that, in itself, aimed to aid students in further concretizing their learnings, critically evaluate their experiences, and build a toolkit of practical wisdom that could be applied to community engagement beyond the fellowship. Responses were available for nine iterations of the fellowship from its inception (Spring 2018 to Spring 2024) for a total of 168 data points, with response rates within each year ranging from 50 to 94% (Table 3). Data from four cohorts (Fall 2019 to Spring 2021) were unavailable due to data-collection-related disruptions associated with the COVID-19 pandemic.

**Table 3:** Distribution of post-graduate public health student and trainee responses to reflective community engaged learning fellowship debrief exercise.

Year	Count of respondent fellows	Total fellow count	Response rate	Count of responses	Percent of all responses (*n* = 168)
2018	16	32	50%	29	17%
2019^1^	16	17	94%	32	19%
2021^2^	11	14	79%	11	6.5%
2022	19	30	63%	23	14%
2023	28	37	76%	55	33%
2024	11	15	73%	18	11%

1 Missing data from Fall 2019 cohort.

2 Missing data from Spring 2021 cohort.

Deidentified responses were analyzed thematically and initially coded separately for both prompts. In the first stage of the coding process, all responses were sorted by grouping those with similar content and language but without necessarily assigning a label, aiming to minimize investigator-imposed assumptions or preemptive interpretations. Two or more distinct ideas within a single response were separated for more precise coding. In the second stage, a combination of descriptive and *in vivo* coding was carried out on grouped responses, which synchronously and inductively informed codebook development for both prompts, including developing code descriptions, quoting examples, and specifying exclusion criteria. Codes were continually refined (e.g., added, separated) and responses recoded based on the updated codebooks, with the primary aim of identifying the latent interpretive frameworks underlying students’ common experiences and meaning-making processes (21). As a third and final stage, codes were combined into one unified codebook (Appendix), again refining codes (e.g., combining) and identifying overarching domains to organize sets of similar codes based on operative level (e.g., personal, community, institutional) until saturation was reached (22).

Themes were developed as central organizing concepts (21), informed inductively by coherent clusters of codes cutting across domains and capturing the overarching schema by which students internalized and matured as public health professionals in their service-learning experiences. Thematic development also aimed to reveal key insights into the values, attitudes, and skills fellows developed during their time in the field as relating to the three main learning objectives of the program: posture, proximity, and positionality. While staged coding was carried out by one coder (with periodic discussions with a second investigator), both investigators reviewed all codes and domains together in real time to inform thematic development. Deidentified quotations from a broad range of fellows exemplified each theme in our findings.

Due to the use of de-identified data and the secondary nature of this analysis, this study was not considered human subjects research and required no formal review from the institutional review board at the Harvard Longwood Campus. Data were de-identified at collection and, as such, contain no demographic information.

## Results

The saturated codebook was composed of 24 codes organized under five domains (Supplementary Table 1). Seven themes arose from the clustering of codes across these domains, identified as follows: under posture, (1) humility, (2) listening to learn, and (3) present and authentic; under proximity, (4) relationality and (5) flexibility; and, under positionality, (6) centering community expertise, and (7) power shifting. Although developed inductively, these themes are presented systematically, organized by the three main learning objectives of the program (i.e., posture, proximity, positionality) and characterized as a value, attitude, or skill to evaluate the fellowship’s effectiveness in guiding student learning experiences in the field within its own pedagogical framework. Quotations are identified by year and prompt answered, with one referring to “What did you wish you knew before your CEL experience?” and two referring to “What advice would you give to the next cohort of fellows?”

### Posture

#### Humility

The theme of humility was central to many fellows’ field experiences and, in particular, surfaced as a value which they imparted in their advice to future cohorts. Fellows acknowledged that there was much to learn outside of the classroom and, more critically, that the tools they were often equipped with in academic settings were not built to learn from community members. For the fellows, embodying humility as a value, therefore, meant taking their “academic” selves less seriously, having the bravery to admit gaps in knowledge, and approaching conversations with the intention to learn, respectfully and with an open mind.

*“Have a sense of awareness: of yourself, as a visitor of the community; of the community members and their perspectives and values; and of the differences between both. Remember to approach conversations with humility, [treat] people with respect, and acknowledge that you are not the expert in the room. Community engagement works best when we listen, learn, and support—not when we try to lead and make all the decisions. […] Be respectful and remember that you are there to listen, not to speak.”* (2019) (2).

In many ways, humility served as the foundation by which fellows took on the posture of a learner in their interactions with individuals and partners in the community. It fostered a disposition that assumed that fellows’ questions should be more numerous than their answers, that community members very often knew more about the public health issue at hand (as well as the challenges, contexts, and complexities surrounding it) than academic experts, and that everyone in the community (not just those designated as a “partner” within host organizations) held knowledge that could be learned from.

#### Listening to learn

Listening, with the explicit intention of learning, was the skill by which many fellows enacted the value of humility. Informal conversations, for example, often served as the hotbed by which fellows had the opportunity to move their projects toward unexpected directions and, more profoundly, practice the openness and flexibility to learn from or serve their community partners in unexpected ways.

*“Listening is your biggest tool. Serving is your end mission.”* (2022) (2).

For fellows, listening to learn meant asking “anyone and everyone” (2024) (2) open-ended questions in order to, in part, better understand the public health problem of interest as well as identify more effective solutions. The skill of listening also entailed the fellows remaining curious, trusting the host organization in guiding their tasks and overall project goals, and following the broader community where it may lead them. Fellows also reflected on the capacity of listening to strengthen bonds they created with community members and further enrich their learning experiences.

#### Present and authentic

To practice the skill of listening and enact the value of humility, fellows expressed the need to engage with their projects and partners with an attitude that prioritized showing up as intentionally present and authentic; demonstrating respect, empathy, and patience toward community members; and practicing iterative reflection, including consistent journaling about their personal experiences. For many fellows, enabling this disposition required being mindful of and/or letting go of formalities associated with their academic institution.

*“Do not be afraid to be ‘unprofessional,’ in the sense that hierarchy, formalities and posturing often only distance us. Let people interact with your most comfortable self; this experience should be reciprocal.”* (2024) (2).

Present authenticity also entailed a desire among several fellows to immerse themselves more fully in the cultural contexts of their host communities, getting involved, for example, by learning a new language, attending community events, or exploring friendships with colleagues and other community members outside of the formalities of the workplace.

*“If you are working in a setting where you are not fluent in the language, take time to learn the language! It shows that you are learning, that you are willing to make mistakes and get corrected, and that you are engaged/committed. It is also a great way to make friends.”* (2018) (2).

An intentional posture of authenticity sometimes entailed adopting a level of vulnerability that challenged fellows to meaningfully engage before “feel[ing] fully ready” (2023) (1), surfaced a difficult sense of being “perceived as an outsider” (2024) (2), or left them overcommitted and in need of rest. Challenges like these left some fellows feeling incompetent or helpless to make the positive contributions they expected to. At the same time, navigating these personal and practical difficulties also helped fellows call into question the frameworks by which they may have come to have inaccurate or incomplete assumptions about their partner communities before entering the field.

### Proximity

#### Relationality

Prioritizing relationship building surfaced as a major value in many fellows’ learning experiences, benefitted explicitly from being proximal to community members and partners in the field. Fellows expressed wishing they had “built in more downtime” (2023) (1) to get to know those working in their partner organizations (e.g., getting lunch). They also described the practical value of taking the time to build a team who could teach and support them, especially in difficult or unexpected times (including relationships with other fellows and fellowship staff). Beyond logistical benefits, however, fellows recognized that relationships often served as the foundation by which their engagement could extend beyond their specific projects, creating meaningful connections and long-term partnerships for longer lasting impact.

*“Connection is the most valuable piece you can take away and an important piece to leave, so be present.”* (2022) (2).

Many fellows recognized that relationship building can take time and, importantly, requires having an attitude that prioritizes trust building without assuming that trust is deserved but, rather, earned. Some students acknowledged that fellows may not be immediately accepted in the community as “having good intentions” (2024) (1) and that, therefore, it was important to show up with sincerity, “humility, and sustained effort” (2024) (1).

*“At first, discard your agenda. Just authentically connect with people and learn their stories. Gain trust, then start [the] work.”* (2024) (2).

#### Flexibility

Being proximal to community partners in the field positioned fellows to approach their work with an attitude of flexibility as centrally important to their community engaged projects. The unexpected challenges that came with establishing relationships, identifying resources, and implementing their projects, for example, were only experienced after having practical opportunities to translate their theories and plans into action. In several instances, specific project tasks (e.g., recruiting participants) took longer than fellows anticipated. In others, internal or external politics around an organization had unexpected influences on project goals. Fellows wished they had known, or advised future fellow to recognize, that “things will change” (2023) (1) and that “adaptability is essential” (2023) (1).

*“I wish I had trained to surrender to the process in reality. (I thought I had a theory.) I caused myself a lot of frustrations because of that futile attempt of control. Things worked out so much better when I just flowed and surrendered.”* (2023) (1).

Importantly, the need to “pivot” (2021) (1) in their project planning helped many fellows recognize the importance of centering the priorities of community members and their partner organizations. It also provided an opportunity for fellows to practice letting go of control, overpreparation, and preconceived project expectations. Instead of simply preparing for logistical hurdles, many fellows came to understand that community engagement was more meaningful when it centered the process more than the outcome. Small tasks identified as needed by the organization (e.g., notetaking, resource coordination), a slowing down of project timelines, or unexpected conversations served as the foundation for more fruitful learning and service experiences.

*“Preparation—in the way students often conceive it to be—is not a recipe for success. Overpreparation will likely distract you from the important work.”* (2019) (1).

*“Listen and be deliberate about what value you can add for the time and resource[s] you are being given. Jump into everything you can and be an extra pair of hands when needed; it will ultimately enhance your learning and project goals.”* (2018) (2).

*“As you move through your project, make space for the unexpected and slow down so you can truly listen and observe.”* (2023) (2).

### Positionality

#### Centering community expertise

Enacting flexibility as an attitude in project goals ultimately served to help fellows recognize centering community expertise as a value essential to community engagement. Fellows described the need to involve their partner organization in project development early on, from identifying priorities to establishing plans for evaluation and feedback loops to inform future programmatic goals. The themes of humility and listening to learn also often surfaced alongside advice to decenter fellows’ own priorities and expertise in deference to that of community members.

*“Do not be too attached to a certain outcome. Rather move at the pace [that is] in alignment [with] the goals and needs of the community. This is their work, and they will sustain it. Help them move forward [with] what feels doable and timely to them.”* (2024) (2).

Storytelling surfaced as a powerful means by which fellows were able to listen to community members more authentically and form genuine relationships with partners. For many fellows, storytelling also served as a form of knowledge generation typically undervalued in academic spaces and, thus, uplifted community expertise and more agentic pathways forward. Importantly, others noted that storytelling could also reify harmful narratives about their partner communities if not shared with a positioned and critical lens.

*“Appreciate the gift of people sharing their story with you, and be grateful for their trust.”* (2023) (2).

*“Be aware of the narrative you take with you.”* (2023) (1).

#### Power shifting

Centering community members’ expertise highlights a specific way in which fellows expressed a broader need to shift power away from themselves and broader systems and toward individuals and communities. Within this framework, however, it was important for some fellows to call into question the generalizations by which communities are described as having homogenous interests—as well as their own prior failures to consider diversity in the perspectives, values, and priorities of individuals within their host communities and organizations.

*“It is important to think broadly and critically about how we discuss ‘community.’ This often is with assumptions about shared needs, experiences, and identity which mask/ignore unique individual perspectives. The ‘community’ is a social construct which can be used dangerously—despite good intentions.”* (2018) (2).

*“Communities are not a monolith! There are intra-community dynamics you may not be aware of. Consider how this impacts your work and findings.”* (2021) (2).

Public health frameworks, which fellows expressed learning didactically, also often approach research and interventions through need- and deficit-based understandings of communities rather than centering agency, strengths, and collective action.

*“Focus on community assets, not deficits. What you see as the community’s needs are likely not what they actually need. A deficit-based model assumes your authority and a lack of community power. But the community has a lot to offer. Bolstering community strength allows us to promote community spaces instead of perpetuating a hierarchical institutional perspective.”* (2023) (2).

Many fellows recognized that their institutional positioning could also carry both positive (e.g., high standards) and negative (e.g., elitism, history of extractive research activity) assumptions within the communities they worked with. Some asserted the importance of ensuring that their community partners’ trust, often associated with such affiliations, was not taken for granted. Ultimately, fellows expressed the need to intentionally ensure that their community engagement truly benefitted their partner communities and did not serve extractive academic interests alone.

## Discussion

Leadership development in public health education hinges upon the inclusion of community engaged, experiential service-learning, a pedagogical approach that instills a set of values, attitudes, and skills that are, ultimately, premised on becoming a “learner.” CEL requires not only a willingness, but a desire to get to know people, learn about the public health issues communities face, and confront the systems and structures that create and sustain them. Beyond these more active and relational elements, CEL also cultivates an interiority that centers reflection and observation, creating the space needed for students to try out new ideas and recognize assumptions that may stand in the way of community-identified priorities, needs, and strengths (23). CEL, more broadly, provides future public health leaders the opportunity to exercise ethical and authentic engagement in a personal, pragmatic, and critical sense.

Our findings speak to the capacity of community engaged service-learning to contribute to a kind of public health practice that deepens dependence on a new set of “Four P’s:” personal growth, place-based learning, authentic partnerships, and transformative power. Through community engagement, students and trainees are invited to truly listen to learn, growing an interior disposition toward humility and flexibility that decenters self-interests, uplifts community expertise, and promotes more collective forms of decision-making. More than just a method, community engaged service-learning is a “way of being,” always seeking out an awareness of self in relation to others and the public health objectives at hand. Because projects take place “in the field” (Supplementary Table 2), service-learning is inherently tied to a specific place, allowing students to immerse themselves in the messiness and challenges that necessitate a more reflexive, inclusive form of problem-solving. These experiences also facilitate the formation of more authentic relationships with community members, built on trust and a willingness to embrace the more contradictory dimensions of partnership as opportunities to exercise adaptability and equitable collaboration. Ultimately, these personal, proximal, and interpersonal dynamics set the stage for learning built on service, transforming conceptualizations of power from those that are blind, individuated, and repressive toward those that are conscious, collective, and reclamatory.

We are intentional, here, in our attempt to reclaim the meaning and intention of “service” in community engaged service-learning. In both theory and practice, formulations of service run the risk of becoming individual acts of volunteerism or charity, blind to root causes and with built-in assumptions around communities as powerless victims, incapable of acting for themselves or for systemic change (24). Yet, to critique service as charity would be to ignore the pedagogical power of charity as a “giving of the self, expecting nothing in return, and with no expectation that any lasting impact will be made” (24). Asking students to serve communities without a broader political agenda runs the risk of their activities ending only in serving particular people in particular places. It also positions service as involving self-formation, prompting students to turn a mirror toward themselves, reflecting on and critiquing what facilitates, and impedes, a decentering of self in their own interior lives, not just in the oppressive systems around them. Yet, in this sense, we argue that supporting students to engage in communities as service-learners brings about the exact kind of transformative learning needed in contemporary public health leadership development: personal growth that is, nonetheless, foundational to, and deeply interconnected with, systemic change. Authentic service positions students as “servants” to the priorities of particular people in particular places. It is a radical form of power shifting, piercing through distant discussions of systems and structures and into the intimate heart of every individual learner. Service challenges students to enact humility, build authentic relationships, and center community expertise. It recognizes that community engagement is not simply about capacity-building or “empowerment,” as if students and systems alone have power and communities do not. Community engagement allows students to take on the posture and proximity by which they relinquish their own priorities, recognize that power already exists in marginalized spaces, and be of service to that power (25), becoming leaders who are public “servants,” in a truer sense of the word.

It does not escape us that practicing humility is a kind of disempowering exercise in and of itself and, as such, can impart a sense of hopelessness or helplessness as students come to terms with the limits of their knowledge, the extent to which they were unable to achieve project goals, the true scale of the public health issues at hand, or how deeply challenging addressing their complexities might be. In our CEL framework, we are, therefore, also intentional about conveying the importance of reclaiming fellow’s own sense of power as public health practitioners, one which we hope becomes more intimately and intentionally tied with community engagement. Power is collective and can be reclaimed in collaboration with and deference to community partners. Systemic change is, indeed, possible, but it most often occurs more justly through shared, community-owned decision-making, reflective of multiple sets of priorities, lived experiences, and expertise.

### Implications

In the era of Public Health 3.0, if not a “servant,” this leadership archetype might be best described as a “strategist,” serving as a connector, building coalitions, and actively collaborating with diverse stakeholders across sectors to develop systems-level approaches to health promotion (26, 27). A recent American Public Health Association policy statement has called for the “reimagining of public health leadership for health equity” and a movement toward community engagement and “collective leadership,” premised on shared power, self-awareness, humility, and life-long learning (28). Instead of an individualized “hero model,” this more inclusive narrative recognizes that public health leadership is the result of the actions of many, but especially that of community members closest to the complexity of social issues and who, therefore, are often best equipped with the strengths and wisdom to enact positive change (29, 30).

Our findings reinforce the value of, and serve as a model for, community engaged service-learning as a pedagogical strategy in public health leadership education and training. When rooted in learning objectives that center taking on the posture of a learner, CEL offers public health students and trainees experiential opportunities to solidify leadership values based in social justice, respect, interdependence, and self-determination (31). Catalyzing public health leadership entails programmatic design in graduate education, in particular, that reclaims service that moves toward a more critical approach. Rather than volunteerism or “do-goodism” in community settings alone, learning objectives in community engaged public health curricula must include the necessary interrogation of position and power that duly recognizes the right and ability of communities to exercise agency in identifying priorities, setting agendas, designating resources, and designing programs implemented in partnership with academic institutions.

### Limitations

The qualitative data in our study were derived from open-ended prompts designed with the primary intention of eliciting continued critical reflection and peer-to-peer knowledge sharing around CEL fellows’ field-based experiences and, as such, served as a secondary means of evaluating student learnings within the program’s pedagogical framework. They were also deidentified and, thus, cannot speak to potential differences in experiences by, for example, department or degree program. Nonetheless, using these data allowed thematic analyses to reflect a more unstructured, inductive assessment of fellows’ learning experiences and, as such, was inclusive of themes that laid beyond our main learning objectives. Along with this qualitative approach, more recent efforts in the program have also incorporated a more formal evaluation of fellows’ learnings within the CEL framework through quantitative, survey-based measures administered online as part of program debrief. The results of this study have also begun to inform program pedagogy, including teaching materials and themes discussed during orientation and debrief. Still, it is important to note that fellows’ responses do not necessarily reflect their translating of stated learnings into practice, both during the fellowship and beyond, pointing to the need for collecting complementary forms of evaluative data, such as those from host organizations or from fellowship alumni who have graduated and now serve in public health leadership roles.

Over 6 years of CEL program development, we have also reflected on, anecdotally, the challenges often associated with the short-term nature of many fellows’ projects, the main tasks of which are most often implemented during a single summer or winter term. As highlighted in the broader literature, there is a pressing need for serving-learning programs to establish structures for more long-term commitments (32), including those that recruit students interested in contributing to an already established, ongoing partnership between their academic institution and a host organization rather than engage in their own partnership development and program design. At Harvard Chan, these efforts are nascent through recent partnerships in the Mississippi Delta. Far from perfect, these processes necessitate program staff and faculty involvement to craft structured yet flexible community engaged service-learning models that center prolonged, co-created initiatives while also accommodating unique student learning interests and needs. Collecting feedback from partner organizations evaluating student engagement and the broader fellowship program itself can facilitate these improvements.

## Conclusion

Community engaged service-learning has the potential to contribute to transformative public health education, promoting leadership skills that foster a deep-rooted commitment to community partnerships while also instilling a more authentic valuing of humility, adaptability, relationality, and interdependence (33). Embracing CEL in graduate education serves as a promising vehicle to ensure future public health leaders can meet the demands of diverse and dynamic challenges in a variety of sectors (e.g., research, policy, advocacy) with competence and compassion. Community engaged service-learning teaches leaders to identify, question, and disrupt power dynamics not only by addressing social determinants of health but, more profoundly, by recognizing and reckoning with assumptions that may contribute to power differentials within their own relationships and spheres of influence. Far from simply bringing awareness to power and positionality, however, community engaged learning encourages students to move forward in spite of these trappings, co-creating solutions in more deferential, and, therefore, more equitable, ways.

## Data Availability

The raw data supporting the conclusions of this article will be made available by the authors, without undue reservation.

## Supplementary material

SUPPLEMENTARY TABLE S1 Analysis codebook.

SUPPLEMENTARY TABLE 2 Examples of CEL projects.

SUPPLEMENTARY DATASHEET 1: Community Engaged Learning Fellowship Program Handbook.
