# Supplementary material for: Posture, proximity, and positionality: the power of community engaged service-learning in public health leadership education
Source: Front Public Health. 2025 Jul 2;13:1605757. doi: 10.3389/fpubh.2025.1605757 (PMC12263936; doi:10.3389/fpubh.2025.1605757)
Supplement: SUPPLEMENTARY TABLE S1 — Analysis codebook. [file Table_1.docx]

**Supplementary Table 1** Reflections from post-graduate public health students and trainees on what they wish they had known before their community engaged learning fellowships and advice for future fellows

| No. | Code | Description/Inclusions | Example(s) ^1^ | Exclusions |
| --- | --- | --- | --- | --- |
| **1** | **Personal formation** |  |  |  |
| *1.1* | *Balancing commitments, rest, and personal growth* | Remaining self-confident and tending to career development and respite during fellowship | “I wish I had known (or prepared) to take more breaks - passion and excitement still need to be replenished with rest.” (2024) (1)  “Be kind to yourself when you make mistakes or are corrected or when tasks are not completed. Have fun and learn.” (2023) (2) |  |
| *1.2* | *Being human to be more present and proximal* | The benefits of “being human” to cultivate a more present, empathetic, and open-minded disposition; includes shaking off formalities associated with position, titles | “Don't be afraid to be ‘unprofessional,’ in the sense that hierarchy, formalities, and posturing often only distance us. Let people interact with your most comfortable self; this experience should be reciprocal.” (2024) (2) |  |
| *1.3* | *Cultivating humility as a learner* | The importance of listening and recognizing community members’ expertise, while decentering your own, as part of the learning experience | “Hopefully, you'll be going in with a willingness to be humble and open-minded, to learn from the community. But take a step forward. Recognize that the tools at your disposal might not be built to learn from the community at all.” (2019) (2) | 4.3 |
| *1.4* | *Centering the process, not the outcome* | Recognizing that service/learning occur throughout the project, not just at the end, and depend on the posture of the learner | “I wish I knew that the process is just as (or more) important as the outcome/product.” (2019) (1) |  |
| 1.4.1 | Diving in / letting go of control | Relinquishing a strong sense of control over project expectations; accepting, on a personal level, that it is difficult to ever feel fully “ready” or prepared | “Meaningful engagement often happens before you feel ready. You don't have to get it perfect - the most important thing is to listen and stay open.” (2023) (1) | 1.4.2, 3.5 |
| 1.4.2 | Leaning into flexibility and learning in the little things | Having the flexibility to learn and serve the community in small, unexpected ways, including those outside of initial project goals | “I wish I knew how important the little things can be in this experience. I quickly learned the importance of observations and small, unexpected conversations.” (2023) (1)  “Setup your plan but remain flexible as some of the most impactful (according to your agenda) events may happen outside of the plans.” (2023) (2) | 3.1 |
| 1.5 | *Practicing iterative reflection* | Thoughtfulness through repeated and ongoing reflection and journaling | “Bring a journal and write about your experiences. Talk to people (everyone) that you encounter and really immerse yourself in the community.” (2023) (2) | 4.3 |
| **2** | **Place-based field considerations** |  |  |  |
| *2.1* | *Immersing yourself in the context and culture* | Cultural immersion as a part of authentic community engagement | “Engage not just with [...] the organization in a formal capacity. Be present more broadly, if they value church, go to church! If they value the outdoors, go hiking! If they value certain food, enjoy with them! Engage as a peer as much as you are accepted this will enrich your understanding and experience and impact.” (2024) (2) |  |
| *2.2* | *Preparing for logistical challenges and language barriers* | Anticipating and learning from challenges in travel, lodging, language, resource availability, and other logistics | “Language can be a real barrier that can impact how you connect with the community and so putting the right measures in place [...] is essential.” (2023) (1)  “I wish I had been more prepared for and aware of the logistical challenges of traveling around the context I was working in. That said, these challenges contributed to a broader lesson and takeaway of my time in the field!” (2023) (1) |  |
| **3** | **Community partnership and relationships** |  |  |  |
| *3.1* | *Listening to community members and getting diverse perspectives* | The act of listening (and getting as many perspectives as possible) as central to community engagement, problem-solving, and relationship building | “Listen, listen, listen to anyone and everyone. Make an effort to learn more than you feel you might need to teach. Stay curious. People will surprise and inspire you if you let them.” (2024) (2)  “Don't limit the amount of people you can learn from.” (2022) (2) | 1.4.2, 3.3, 3.4 |
| *3.2* | *Prioritizing relationships* | Centering relationships as among the most valuable aspects of the project/partnership | “Make sure to have a supportive team with you. They will serve you well in tough times.” (2023) (1)  “Connection is the most valuable piece you can take away and an important piece to leave, so be present.” (2022) (2) | 3.5.2, 3.3 |
| *3.3* | *Taking the time to build trust* | Having the persistence to build trust as the bedrock for collaboration, relationships, storytelling, and effective work | “Budget and be thoughtful for how you build relationships - it will require humility and sustained effort.” (2024) (1)  “Small steps at a time to build trust by interacting with people. Showing your passion to listen to community's voice, voice might be small. But once you identify it, it might change the community and life.” (2024) (2) |  |
| *3.4* | *Planning ahead and involving community partners before, during, and after project end* | The benefits of patient, preemptive and ongoing planning, including communication with partner organization, to inform project development and make the most of limited time in the field | “To involve the community, you will want to work from the beginning (drafting your project) to the end (analyzing findings and disseminating research findings).” (2023) (1)  “It will end so plan a transition and exit strategy.” (2019) (1)  “Get clear on your goals for the summer and your partner organization's goals. Inevitably, time will go faster than you anticipate and with both your and your organization's goals written out, you will be better equipped to decide what to prioritize.” (2023) (2)  “Be patient. Although your project is a #1 priority for you, the organization has 20 other urgent topics they are working on.” (2018) (2) | 3.1, 3.5 |
| *3.5* | *Responding to unexpected changes in project plans* | Expecting and managing the unexpected in project implementation | “Be prepared but aware of possible detours. Things will change.” (2023) (1) |  |
| 3.5.1 | Prioritizing personal learning | Problem-solving with largely personal goals (e.g., project, learning) in mind | “Your project will change, and you should have goals in place to support your learning. The time goes by quickly, so if you have a plan to deviate from and lean into community engagement, you will get more out of your time.” (2023) (1) |  |
| 3.5.2 | Prioritizing relationships | Problem-solving with mainly relationships (e.g., community partners) in mind | “Relax, be flexible, and prioritize building relationships and adding value.” (2018) (1) |  |
| 3.5.3 | Prioritizing community partner goals | Problem-solving with largely community partners’ priorities in mind | “Your plan will likely change once you get ‘into the field,’ which is not only okay, but often the right thing because it means you are taking time to understand, listen, and adapt based on what you learn from that.” (2019) (1) |  |
| *3.6* | *Serving the community / giving back* | Balancing projects goals or personal learning with authentic service | “Make sure what you do, the community benefits, and make sure to communicate (via dissemination and engagement) so you are not just extracting and giving back.” (2018) (2) |  |
| **4** | **Power and institutional dynamics** |  |  |  |
| *4.1* | *Grappling with institutional reputation* | The impact, benefits, and challenges of coming from an academic institution, and reflections on awareness and management | “It can feel challenging to be an outsider, and relationship building takes time! Coming under the ‘Harvard name’ carries a lot of assumptions (both good and bad).” (2024) (1) | 1.2 |
| *4.2* | *Recognizing power dynamics and challenging overgeneralizations* | Bringing awareness to, and deconstructing, power dynamics around the monolithic concept of “community” | “Never underestimate the power of politics (internal and external) to get [in] the way of what a community wants and/or needs.” (2019) (1)  “It's important to think broadly and critically about how we discuss ‘community.’ This often is with assumptions about shared needs, experiences, and identity which mask/ignore unique individual perspectives. The ‘community’ is a social construct which can be used dangerously - despite good intentions.” (2018) (2) | 3.1 |
| *4.3* | *Challenging assumptions and recognizing community agency* | The importance of interrogating positionality and personal assumptions, including deficit-based perceptions of community needs and priorities | “Any assumptions or beliefs or generalizations about the community are wrong.” (2024) (1)  “Do not hold onto your idea too tightly. Let the community openly interrogate, scrutinize, and question your ideas/thoughts.” (2023) (2)  “Never do work as an act of charity. Give people respect and treat them as an equal person.” (2023) (2) |  |
| *4.4* | *Uplifting the power of storytelling* | The impact, meaningfulness, and pitfalls of narratives/storytelling | “Behind the ‘participant,’ the ‘recipient,’ ‘beneficiary,’ and ‘coordinator,’ are individuals with a story and resilience.” (2023) (1)  “Be aware of the narrative you take with you.” (2023) (1) |  |
| **5** | **Fellowship experience** |  |  |  |
| *5.1* | *Clarifying service learning with fellowship/partners* | Confusion about the meaning of service learning in a community engaged project | “[I wish I knew h]ow to work with host/partner organizations (who may have different expectations) to build a service learning project.” (2018) (1) |  |
| *5.2* | *Getting to know other fellows and leaning on fellowship support* | Desire to form stronger relationships and support systems with other fellows and fellowship staff | “I wish I had a stronger understanding of the other projects in my cohort and developed stronger relationships with others who I didn't know before to be more connected during the summer.” (2018) (1)  “Ask for help or support if you need it! RSLF is ready to help!” (2018) (1) |  |
| ^1^ Numeration identifies the year and prompt answered by the quoted response, with one referring to “What did you wish you knew before your community engaged learning experience?” and two referring to “What advice would you give to the next cohort of fellows?” | | | | |
