# Supplementary material for: Posture, proximity, and positionality: the power of community engaged service-learning in public health leadership education
Source: Front Public Health. 2025 Jul 2;13:1605757. doi: 10.3389/fpubh.2025.1605757 (PMC12263936; doi:10.3389/fpubh.2025.1605757)
Supplement: SUPPLEMENTARY TABLE 2 — Examples of CEL projects. [file Table_2.docx]

**Supplementary Table 2** Recent examples of community engaged learning fellows’ projects at Harvard T.H. Chan School of Public Health by degree program and project location

| Semester and Year | Project title and partner organization | Location | Degree program | Department(s) or Field(s) of study |
| --- | --- | --- | --- | --- |
| Fall 2024 | Increasing employability for adults with Down Syndrome  *Down Syndrome Association of Toronto* | Toronto, Canada | Master of Science | Epidemiology |
| Fall 2024 | Occupational safety and health practices in small-scale mining in rural Ghana  *Cocoa360* | Tarkwa-Breman, Ghana | Master of Public Health | Environmental Health |
| Fall 2024 | Promoting healthy lifestyles through revitalizing traditional foods in Ecuador  *Asiri Health Arts* | Sangolqui, Ecuador | Master of Public Health | Health Management |
| Spring 2024 | Challenges in coastal vulnerability for Qingdao's offshore aquaculture  *Yellow Sea Fisheries Research Institute, Chinese Academy of Fishery Sciences* | Quindao, China | Master of Science | Environmental Health |
| Spring 2024 | The role of food vendors in school environments: A qualitative study in Tanzania  *Africa Academy for Public Health* | Dar es Salaam, Tanzania | Master of Public Health | Nutrition |
| Fall 2023 | Improving emergency birth skills among midwives at Jinja Hospital, Uganda  *Jinja Regional Referral Hospital* | Jinja, Uganda | Master of Public Health | Global Health, Social and Behavioral Sciences |
| Fall 2023 | Mental health awareness through an art-based protocol  *NeuroDiseño Humano* | Quito, Ecuador | Master of Public Health | Health Management |
| Spring 2023 | Engaging the “hard-to-reach:” Examining the barriers to HIV testing and care  *Mbarara University of Science and Technology & Weill Cornell Medicine* | Mbarara, Uganda | Master Science | Global Health and Population |
| Spring 2023 | “The Family Van:” Caring for the Boston community  *The Family Van* | Boston, USA | Master of Science | Epidemiology |
| Fall 2022 | Gaps and opportunities for improved water practices in Intibucá, Honduras  *Shoulder to Shoulder, Inc.* | Intibucá, Honduras | Master of Public Health | Global Health |
| Spring 2022 | Public health for the people: A community-centered education program  *Allston Brighton Health Collaborative* | Boston, MA | PhD | Social and Behavioral Sciences |
