# Supplementary material for: Posture, proximity, and positionality: the power of community engaged service-learning in public health leadership education
Source: Front Public Health. 2025 Jul 2;13:1605757. doi: 10.3389/fpubh.2025.1605757 (PMC12263936; doi:10.3389/fpubh.2025.1605757)
Supplement: SUPPLEMENTARY DATASHEET — 1: Community Engaged Learning Fellowship Program Handbook. [file Data_Sheet_1.pdf]

Harvard T.H. Chan

COMMUNITY ENGAGED LEARNING

The Community Engaged Learning Fellowships Program Principles and Practice

HARVARD T.H. CHAN SCHOOL OF PUBLIC HEALTH
Office of Field Education and Practice

Community Engaged Learning

Table of Contents

01 Overview and Purpose

02 Community Engaged Learning Fellowships
- Rose Service Learning Fellowship
- Herbert S. Winokur, Jr. Fellowship in Public Health for the Mississippi Delta
- Global Mental Health Fellowship

03 The Community Engaged Learning Framework

04 The Fellowship Program
- Program Theory, Components, and Learning Objectives
- Writing / Journaling Prompts

05 Resources for Further Learning
- References
- Further Reading

Overview and Purpose

The complex nature of public health calls for deep and continued engagement with communities most impacted by its challenges. Thus, **public health research and practice** requires scholars and practitioners alike to obtain the skills and experience necessary to collaborate equitably and effectively with a wide range of stakeholders directly involved in addressing issues that impact their communities' health and wellbeing. **Public health education**, in turn, has a unique imperative to offer students field-based learning and training in community engagement (Levin et al., 2021).

At the Harvard Chan School, community engaged learning and scholarship serve an essential role in public health education, offering students an opportunity to exercise ethical and authentic engagement with community partners and stakeholders. Based on a service learning framework (Furco, 1996), students engage in their applied practice and research endeavors and learn to listen, reflect, and pay close attention to contexts and social systems – skills that are paramount to careers in public health practice and leadership.

Community engaged learning opportunities:

- respond to and are designed around community-identified priorities and concerns;
- are implemented and evaluated collaboratively and in place;
- balance the service provided to communities and the learning that takes place; and
- create space for ongoing critical reflection, examining social issues by situating the self within a community setting.

The purpose of this handbook is to:

- **Articulate** the rationale and theoretical foundations of community engaged learning in public health education
- **Position** community engaged learning as essential to promoting health equity, social justice, and social responsibility
- **Describe** the programmatic components and pedagogical principles of community engaged learning
- **Provide** a practical guide for implementing a community engaged learning fellowship program within, and as a complement to, broader public health education

Community Engaged Learning Fellowships

Community Engaged Learning (CEL) Fellowships at HSPH support students and postdoctoral fellows across the school and in all degree programs to be involved in community engaged research and practice. These field-based opportunities offer fellows practice in centering community-identified priorities and grappling with issues around power and positionality as they develop projects. Through a cohort-based model, fellows are also invited to initiate new connections with peers to share observations, questions, and challenges and to reflect collectively on their approaches to engaged research and practice.

The Office of Educational Programs offers three fellowship opportunities for students interested in community engaged learning: the Rose Service Learning Fellowship, the Herbert S. Winokur, Jr. Fellowship in Public Health for the Mississippi Delta, and the Global Mental Health Fellowship. Together, the CEL Fellowships aim to:

1. **Address** community-identified needs, working in partnership with community members and partner organizations
2. **Cultivate** a community-centered approach to public health research and practice, including a deeper commitment to addressing structural determinants of health
3. **Develop and strengthen** reciprocal relationships between HSPH and community organizations in which engaged learning projects take place

What are differences between the CEL Fellowships?

The **Rose Service Learning Fellowship** provides funding support for students and postdoctoral fellows to travel and engage in service learning projects. Projects are co-designed with the hosting community partner. This fellowship is awarded in the Fall and Spring annually.

The **Herbert S. Winokur, Jr. Fellowship in Public Health for the Mississippi Delta** and the **Global Mental Health Fellowship** are summer field practice opportunities with established partnering organizations. Winokur Fellows are based in Mississippi and work on projects that address health and economic development in the Mississippi Delta region. Global Mental Health Fellows are based in southern Uganda and work with the John C. Kelly Clinic in Kabale.

The scope of work for individual Winokur and Global Mental Health fellows is co-developed through meetings and conversations between prospective applicants, program staff, and partnering organizations. In contrast, the Rose Fellowship requires applicants to negotiate the scope of projects with a partner organization of their choice and work independently to submit a proposal, workplan, and budget that is competitively reviewed for funding.

How are the fellowships similar?

Fellows form a learning community through the CEL Fellowships Program where they are introduced to the program's framework and commit to community engaged learning principles and practices.

Community
Engaged Learning

Framework

Community engaged learning combines the practice of community engagement with an intention to take the role of a "learner" in the field.

More commonly referred to as "service learning" or "experiential education," this type of pedagogy entails the practice of applying skills and knowledge acquired from the classroom by working in collaboration with a community partner. Distinct from internships, community service, or volunteerism, **community engaged learning moves toward a more "critical approach,"** one that emphasizes the redistribution of power among all members of the engaged learning relationship, development of authentic relationships in the classroom and in the community, and work from a social change perspective.

Mitchell, 2008

Community engaged learning requires:

- A willingness and desire to get to know people,
- Learning about the issues communities face and the systems and structures that create and sustain them,
- Acknowledging our own identities and the identities of those we meet in the community,
- Challenging our own assumptions and beliefs, and
- Recognizing concerns and challenges, as well as strengths and assets.

Donahue and Plaxton-Moore, 2018

Learning takes place through iterative cycles of action and critical reflection.

Community
Engaged Learning

Program

Community Engaged Learning Fellows are part of Learning Communities, both at School and in the Field

As a comprehensive program, CEL fellows commit to participating actively in preparing for and reflecting on community engagement, both with a **community of learners** in the fellowship as well as **communities of practice** in the field. Learning begins at orientation, iterates in the field, and is reflected on during a debrief as fellows return to school.

ORIENTATION
The **principles** of community engaged learning

IN THE FIELD
The **practice** of community engaged learning

DEBRIEF
The **processing** of community engaged learning

CRITICAL REFLECTION.

PEER-TO-PEER LEARNING.

KNOWLEDGE SHARING.

Community
Engaged Learning

Learning Objectives

ORIENTATION

- Take on the **posture** of a learner and seek **proximity** to people and contexts
- Practice **critical reflection,** unearthing personal assumptions and **positionality**
- Consider **multiple ways of knowing**, including **storytelling** as a scientific approach

IN THE FIELD

- Learn as an **iterative practice** of reflection and action
- **Pause to reflect** on sense-making with writing and journaling exercises
- **Share knowledge** with community of learners through web-based posts

DEBRIEF

- Tell stories of community engaged learning experiences and **retell stories** with power and community
- **Make meaning** of events and experiences for **transformative learning**
- Share **lessons learned** in the field with future fellows

01 Situating the Self
The Three P's

Community engaged learning invites fellows to take on the **posture** of a learner, including a willingness to be **proximal** to those most impacted by health disparities. In situating the self within community, fellows are also encouraged to consider their **positionality**, or how their intersecting identities shape how they understand and engage with their projects. Practicing **critical reflection** underpins each of these self-explorations and is emphasized to help fellows challenge their assumptions and engage more equitably with community members while also make personal meaning of their learning experiences.

POSITIONALITY

to understand how our social identities shape our meaning-making experiences and interpretations, as well as those of our community partners

POSTURE

of a learner - meaningful, deliberate engagement, listening to learn, with community partners as equal experts

PROXIMITY

to communities - we cannot learn adequately without getting close, traveling to, immersing in, engaging in organic conversations

CRITICAL REFLECTION

as a practice and as a learning tool to make sense of experiences and learn further about the self as you interact with issues at hand

Community Engaged
Learning Fellowships

What is **your** story?

What brings you to these **people** and to this **place**?
Why this public health issue?

Who are you **here?**

What is your strongest **identity**? How does it **challenge** your engagement with this issue and in this community?

02 Situating the Context **Systems & Action**

Beyond interiority, critical reflection, as an extension of "critical thinking," also asks us to interrogate our ideas of public health issues within broader social, political, and cultural systems. By identifying **structural determinants** and **power dynamics**, we can make more explicit our collaborative and active efforts to disrupt health inequities with communities. After an impactful moment in the field, we can ask ourselves:

**What**	What happened (or is happening)?
**Why**	What is happening below the surface? How did it come to be?
**What Now**	What needs to change? What role do I play in changing it?

Community Engaged
Learning Fellowships

What are the **systems**?

Across your projects, what are **common** underlying power structures influencing the public health issues of interest?

Are you sure?

Given your conversations and experiences in the field, how have your perceptions of these systems **changed**?

03 Situating the Approach

Diverse Ways of Knowing

Exploring the **self**, the **systems** in which communities experience public health issues, and the **solutions** we hope to enact is an iterative and transformative learning process.

Learning through community engaged research and practice is an example of how we generate knowledge in diverse and unexpected ways.

As students and postdocs, fellows come from contexts and with underlying assumptions that might favor academic knowledge generation. In the field, however, redistributing power to our community partners entails recognizing **multiple and diverse ways of knowing.**

Practical

Artistic

Theoretical

Spiritual

Community Engaged
Learning Fellowships

How have you learned?

Tell a story about a **moment** of transformative learning you experienced during your time in the field. Who taught you? Who learned with you? How did you learn?

04 Retelling the Story
Science as Storytelling

Telling stories is a familiar yet powerful way by which we communicate and make meaning out of our experiences. Importantly, in community engaged public health research and practice, stories also enable the redistribution and reclamation of power - away from "evidence-based" academic assumptions and towards an evidence base built on local expertise.

As community engaged fellows, telling our own stories is important, but it can also limits us to our own perceptions. To listen to and uplift others' stories is to humbly liberate truth-seeking and allow it to take on other more powerful and collective forms. Stories are reflexive, thematic, and iterative, teaching us to understand and act in ways we might not have ever imagined.

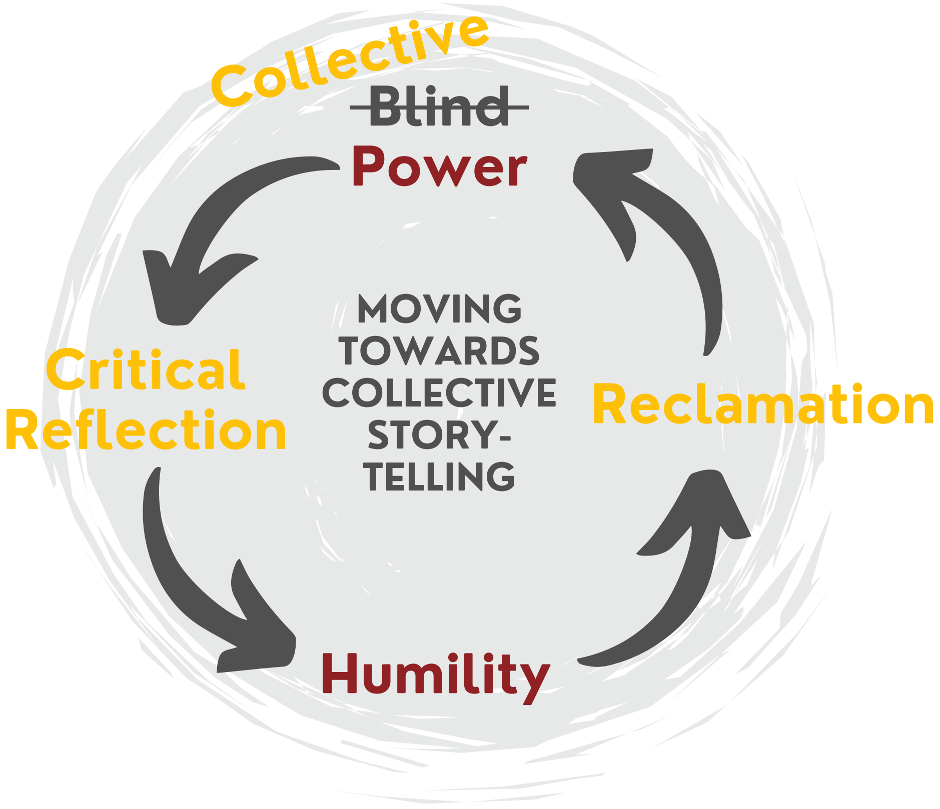

Community Engaged
Learning Fellowships

What is **their** story?

Interview someone you learned from and were close to during your time in the field. What is the public health issues, in their eyes? What are their priorities? What changes do they wish to see?

Community Engaged
Learning Fellowships

Notes

Community Engaged
Learning Fellowships

Resources for Further Learning

References

Donahue, DM and Plaxton-Moore, S. The Student Companion to Community-Engaged Learning. Stylus Publishing, 2018.

Furco, Andrew, Service-Learning: A Balanced Approach to Experiential Education. (1996). Service Learning, General. 128. https://digitalcommons.unomaha.edu/slceslgen/128

Levin, MB., Bowie, JV, Ragsdale, SK. Gawad, AL., Cooper, LA. and Sharfstein, J. Enhancing Community Engagement by Schools and Programs of Public Health in the United States (April 1, 2021). Annual Review of Public Health, Vol. 42, pp. 405-421.

Mitchell, T. Traditional vs Critical Service-Learning: Engaging the Literature to Differentiate Two Models. Michigan Journal of Community Service Learning, Spring 2008, 50-65.

A “Critical” Reflection Framework. Department of Education, Victoria, Australia. https://www.education.vic.gov.au/Documents/childhood/professionals/support/reffram.pdf

What is Critical Reflection? Introducing the “What, So What, Now What” Model. University of Guelph. https://www.youtube.com/watch?v=vGyjF9Ngd8Y

HARVARD T.H. CHAN | SCHOOL OF PUBLIC HEALTH
Office of Field Education and Practice

Harvard T.H. Chan

Office of Field Education and Practice

Community Engaged Learning Fellowships Program: Principles and Practice

This version was published on 4.22.2024

This handbook was developed by Jocelyn Chu ScD, MPH and Abrania Marrero, PhD, SM and designed by Jennifer Weeks, MPH candidate.
